# How Gender Influenced the Experience of Using a mHealth Intervention in Rural Mozambique: Secondary Qualitative Analysis of Community Health Worker Survey Data

**DOI:** 10.3389/fgwh.2022.661000

**Published:** 2022-02-24

**Authors:** Mai-Lei Woo Kinshella, Helena Boene, Esperança Sevene, Anifa Valá, Sumedha Sharma, Marianne Vidler, Laura A. Magee, Peter von Dadelszen, Khátia Munguambe, Beth A. Payne

**Affiliations:** ^1^Department of Obstetrics and Gynaecology, University of British Columbia, Vancouver, BC, Canada; ^2^Centro de Investigação em Saúde de Manhiça, Manhiça, Mozambique; ^3^Faculdade de Medicina, Universidade Eduardo Mondlane, Maputo, Mozambique; ^4^Department of Women and Children's Health, King's College London, London, United Kingdom

**Keywords:** PIERS on the Move (POM), mHealth, community health workers, gender analysis, Mozambique

## Abstract

**Background:**

The mixed-gender community health worker (CHW) program in Mozambique is a window into the different experiences that male and female CHWs may face in their work. The objective of this study is to investigate how gender influenced the experiences of community health workers using the PIERS on the Move (POM) mHealth app in Mozambique.

**Methods:**

This is a secondary analysis by gender of health care workers involved in the Mozambique Community Level Intervention for Pre-eclampsia (CLIP) cluster randomized trial (NCT01911494). A structured survey with 10 open-ended questions was used to elicit CHW experiences using the POM app. Data collection took place in 2017 after completion of the CLIP trial. This analysis examined emergent themes to consider how experiences may have been shaped by health worker gender.

**Results:**

Of the 43 CHWs who used the POM app, there were 31 (72%) women and 12 (28%) men. Gender differences emerged in descriptions of how using POM increased their value and respect by pregnant women and community members. Fifty-eight percent of female CHWs (18/31) said that POM positively influenced their status in the community in comparison to 33% of their male counterparts (4/12). While the small sample sizes, particularly of male CHWs who used POM, preclude conclusions, these findings were supported by qualitative results. Female CHWs tended to elaborate more about community perceptions of their increased value and status as health care providers than male CHWs.

**Conclusion:**

CHWs work within existing gender norms. While gender norms are perceived to support the comfort of women to speak to another woman about their maternal and child health issues, gender norms also work against female CHWs as their professionalism may be questioned more than for their male counterparts. CHW's narratives suggested that the mHealth intervention was valued beyond the technology itself because it also added symbolic clinical value and demonstrated a tangible investment in their professional capacities, which may have been especially appreciated by the female CHWs.

## Introduction

Gender equality is highlighted among the Sustainable Development Goals (SDGs) as fundamental to well-being at all ages (SDG 3) (1). Digital health holds potential to be gender transformative through improved health-seeking among women as well as supporting the skills, social status and effectiveness of community health workers (CHWs) (2). CHWs are a crucial force in expanding health services particularly in rural settings in low- and middle-income countries (LMICs). Mobile health (mHealth) platforms have been deployed to support CHW communication, disease monitoring and the provision of health services (3, 4). However, how digital health influences gendered social relations and promotes gender equality for both clients and providers is often overlooked (2). CHWs in LMICs are often women, they are notoriously underpaid or volunteer and little attention is paid to their experiences using mHealth and gendered experiences in their communities (3–5). Ved and colleagues note that no comprehensive gender disaggregated data exists for CHW programs and several countries have CHW programs that are all female by design, such as in Pakistan and India (5). The mixed-gender program in Mozambique is a window into the different experiences that male and female community health workers face.

Known as *Agentes Polivalentes Elementares* (APEs), the CHW program was introduced in Mozambique in 1978 to strengthen primary health care in rural communities with low health facility density (6, 7). APEs are selected from their communities and live in the communities that they serve (6). APE activities are focused on health promotion and disease prevention (7). The APE program revitalization in 2010 added more curative activities including management of malaria, pneumonia and diarrhea, particularly in children under five (7). The Mozambique Community Level Intervention for Pre-eclampsia (CLIP) (NCT01911494) cluster randomized trial from 2015 to 2017 strengthened capacity of CHWs to detect and manage pregnancy hypertension, a leading cause of maternal mortality (8). Together with community engagement sessions to raise awareness of pregnancy complications and promote birth preparedness and emergency readiness, CHWs in the CLIP trial were trained to use the PIERS on the Move (POM) mHealth app. The POM app guided them through clinical activities for pre-eclampsia identification and management, including measuring blood pressure, dipstick urine test for proteinuria and danger signs to assess pregnant women during home-based care. The POM app calculated risk from these clinical assessments to provide recommendations appropriate to the woman's condition to be delivered by CHWs, including treatment with oral antihypertensive medications, magnesium sulfate (MgSO_4_) intramuscular injection, and/or referral to the nearest health facility (9, 10).

The objective of this secondary analysis of qualitative data is to investigate how gender influenced the experiences of community health workers using the PIERS on the Move mHealth app in Mozambique to identify and manage women at risk of adverse outcomes from pre-eclampsia.

## Methodology

The study is reported according to the Standards for Reporting Qualitative Research (SRQR) (11). The SRQR checklist is included as Supplementary Table 2.

### Study Design and Setting

In this exploratory qualitative study, we undertook a secondary analysis of a cross-sectional evaluation of health care workers in the CLIP Mozambique trial in Maputo and Gaza provinces. Located in southern Mozambique, the study area is largely rural and transport between communities and local health facilities is a recognized challenge (12–14). We followed a constructivist grounded theory approach to inductively explore the meanings CHWs construct within social interactions with pregnant women, their families, community members and other health workers during the CLIP trial while using POM (15, 16).

### Study Participants and Recruitment

All of the CHWs and nurses involved in maternal and child care at primary health centers in the study areas were invited to participate in the health care worker evaluation after the CLIP trial completed. Three local research assistants with no prior personal relationship with participants reached out to CHWs and nurses by phone to introduce the study and to ascertain their interest in participating. Seventeen of the 135 CHWs in the study area were unable to be reached because their phone numbers changed since the trial; all of the eligible participants reached agreed to participate. Sample size of sub-groups was guided by Guest et al's argument that data saturation is often achieved by 12 interviews (17).

### Data Collection

Data collection for the health care worker evaluation took place between October and November 2017, 8 months after completion of the CLIP trial, led by members of the research team (HB, AL) from the Manhiça Health Research Centre (Centro de Investigação em Saúde de Manhiça, CISM). The overall health care worker evaluation included a structured survey that evaluated CHW and nurse confidence regarding their knowledge and skills on the management of pregnant women using 35 questions ranked on a five-point Likert scale administered to all health care workers in the study. For health workers in intervention clusters only, an additional qualitative component was administered consisting of 10 open-ended questions eliciting experiences using the mHealth app. The qualitative component asked respondents for their perspectives and examples of any behavioral changes due to their use of POM, any benefits they experienced from using the POM app and any additional comments on POM or the study. Questionnaires were administered in person in Portuguese or the local Xichangana language, at their homes or in the health facilities where they worked, depending on participant's preference, and lasted an average of 20 min.

### Data Analysis

Participant responses were entered verbatim directly in study tablets in Portuguese by CISM researchers and translated into English prior to analysis. Primary analysis aimed to evaluate the usability and acceptability of the POM application and the impact of implementing POM on CHW's knowledge and self-efficacy to care for women with pre-eclampsia in their communities (18). Primary analysis involved computing summary statistics of demographic characteristics and the responses to the structured survey, including calculating the response average on the 5-point Likert scale and proportions responding within each category. Primary analysis also involved qualitative content analysis on the responses to open-ended questions to explore emergent issues highlighted by participants (19). A coding framework was constructed from emergent categories and transcripts were independently double coded on NVivo 12 (QSR International, Melbourne, Australia) by a Canadian (MWK) and Mozambican (HB) social scientist until there was complete agreement on coding. More information on the primary analyses and qualitative coding framework are reported elsewhere (18).

Of note, the secondary analysis reported in this paper focused on the four codes relating to CHWs out of the larger coding framework in the qualitative dataset, which underwent re-analysis by gender:

- **Knowledge—**What did they learn? This included knowledge gained in general as well as learning about pre-eclampsia, urine testing, how to measure blood pressure, pregnancy care and complications and learning about working with their communities.
- **Self-efficacy**—What did they do with the knowledge and how did it effect their ability to deliver services? This included increased confidence and skills in delivering services, using the skills learned and their ability to inject MgSo4.
- **Empowerment**—How did this change how they saw themselves? This included realizations of their ability to help women in their communities, the importance of their roles and responsibilities and any changes mentioned in self-identity.
- **Relationships**—How did this improve relationships with others? This included mentions of improved relationships with pregnant women, community members and nurses including reports of increased value by others in their services and respect from others as well as how this may have translated into improved quality of care.

Secondary analysis to consider how experiences were shaped by gender involved three components. First, demographic characteristics were summarized by gender on Excel (Microsoft Corporation, Redmond, USA). Secondly, the four codes and their sub-codes described above were enumerated by male and female CHW respondents to better understand their distribution within the qualitative dataset as outlined by Onwuegbuzie and Teddlie (20). Third, direct quotes were drawn to compile narrative case examples. Validity of analysis was enhanced by collaborative analysis and interpretation by Mozambican and Canadian social scientists (MWK, HB, MV, KM, BP), triangulation between the three components of the secondary analysis and use of illustrative quotes to exemplify experiences in their words.

While it is acknowledged that gender is socially constructed and include non-binary forms of expression (2), our analysis explored experiences of self-identified male and female respondents as asked in the survey.

### Ethical Considerations

Ethical approval for the study was granted by the CISM Institutional Review Board in Mozambique (CIBS- CISM/08/2013) and the UBC C&W Research Ethics Board in Canada (H12-00132). Participants provided informed consent prior to data collection. All names in narratives are pseudonyms to protect confidentiality.

## Results

### Demographic Characteristics

Of the 43 CHWs who used the mHealth app, there were 31 (72%) women and 12 (28%) men. This was similar to the proportion of female (85/116, 73%) and male (31/116, 27%) CHWs within the overall Mozambique CLIP trial.

Age and years of experience as a CHW were similar between female and male respondents (see Table 1).The median age of female CHWs was 40 [interquartile range (IQR) 29, 49] and 39 [IQR 33,47] among male CHWs. Years of experience as a CHW were also similar between women and men. The median years of experience was five [IQR 3, 14] among female CHWs and four [IQR 3, 6] among male CHWs who used the POM app. There was a lot of variability within each gender group. Male CHWs who used POM appeared to have higher education levels than their female counterparts, though information was missing in a substantial proportion of respondents (12/43, 28%). Almost all of the male CHWs who used POM completed lower primary education or higher (11/12, 92%) while the rate was lower among female CHWs (20/31, 65%). Male CHWs were also more likely to be married or in civil union (10/12, 83% male CHWs vs. 13/31, 42% female CHWs). In contrast, 17% (2/12) male CHWs were either single or widowed compared to 58% (18/31) female CHWs.

**Table 1:** Demographic characteristics of CHWs that used POM by gender.

		**Overall CHWs**	**Female CHWs**	**Male CHWs**
		**(*n* = 43)**	**(*n* = 31)**	**(*n* = 12)**
Age (years)	Median (IQR)	40 (31, 49)	40 (29, 49)	39 (33, 47)
	Range	22 to 72	23 to 59	22 to 72
Years of experience as CHW	Median (IQR)	4 (3, 6)	5 (3, 14)	4 (3, 6)
	Range	2 to 33	2 to 33	2 to 9
Education	Completed lower primary (5th grade)	6 (14%)	4 (13%)	2 (17%)
	Completed upper primary (7th grade)	16 (37%)	10 (32%)	6 (50%)
	Completed lower secondary (10th grade)	8 (19%)	6 (19%)	2 (17%)
	Completed pre-university (12th grade)	1 (2%)	0 (0%)	1 (8%)
	Missing information	12 (28%)	11 (36%)	1 (8%)
Marital status	Single	13 (30%)	12 (39%)	1 (8%)
	Married/civil union	23 (54%)	13 (42%)	10 (83%)
	Divorced	0 (0%)	0 (0%)	0 (0%)
	Separated	2 (5%)	2 (7%)	0 (0%)
	Widow	5 (11%)	4 (13%)	1 (8%)

### Gendered Experiences of Using the mHealth App

**Figure 1:**
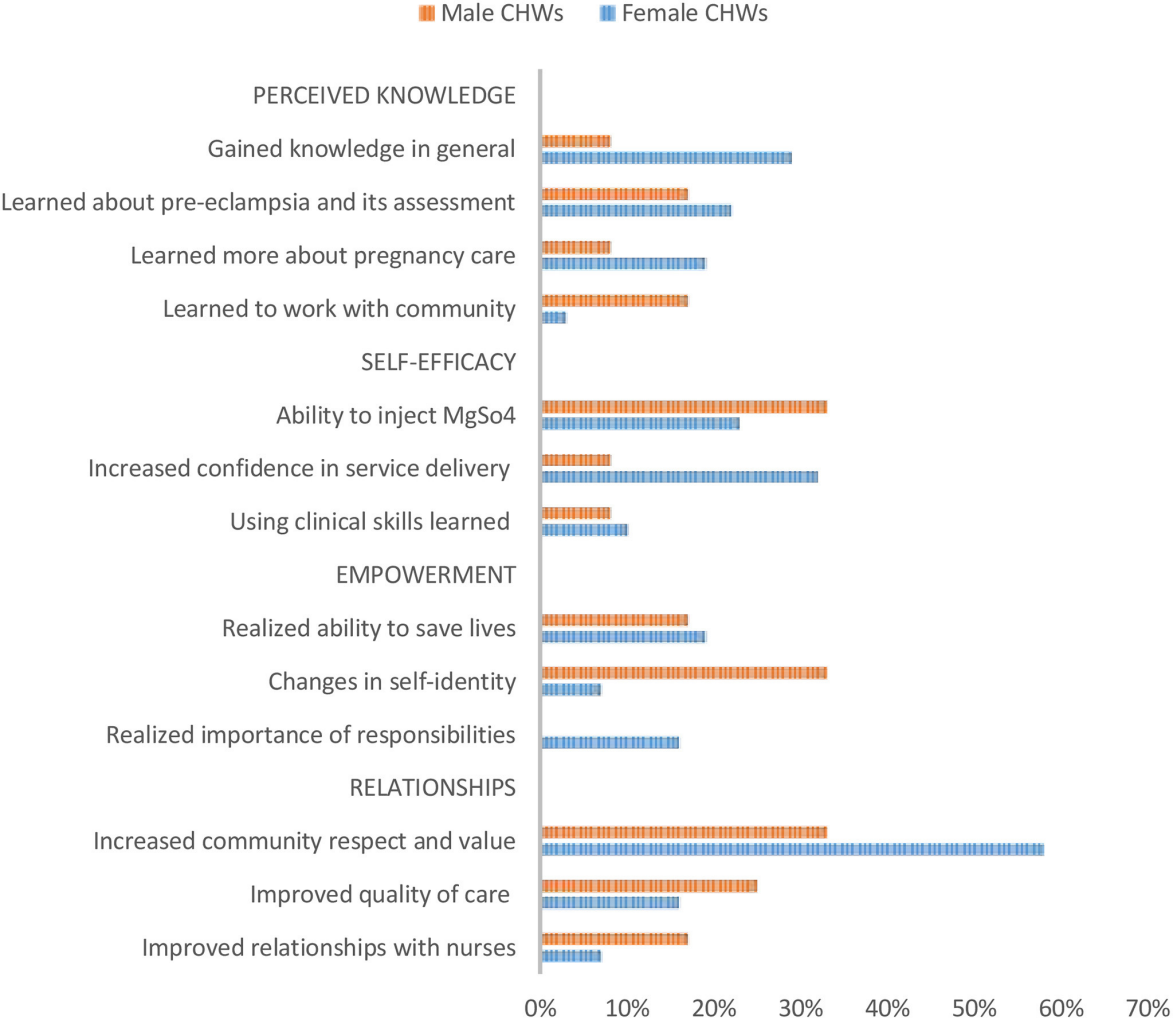
Relative proportion of respondents mentioning key themes to describe impact of using mHealth app by gender.

Across self-reported impact of using POM on CHW knowledge, self-efficacy, empowerment and relationships, different frequency of themes and content of issues raised may be suggestive of how gender may shape experiences of using the mHealth app. These themes are illustrated and described through direct quotes and narrative case examples below. Figure 1 illustrates the proportion of CHW in each gender group who mentioned the themes (see also Supplementary Table 3). Frequency is indicated along with illustrative narrative case examples below.

#### Knowledge and Self-Efficacy

Female CHWs more frequently reported gaining knowledge in the process of using POM (see Figure 1). In contrast to 33% (4/12) male CHWs that shared they had increased their knowledge, 61% (19/31) female CHWs said the same, including more frequent mentions of gaining general knowledge and more specifically about pregnancy care, complications and pre-eclampsia. While male and female CHWs had a similar number of comments regarding self-efficacy, female CHWs more frequently highlighted an increase in confidence and skills in delivering services (10/31 female vs. 1/12 male).

For example, Eliza is a 40-year old CHW with 5 years of experience working in Gaza province who had completed upper primary school. She is a single mother with two children. Speaking about gaining knowledge about pregnancy care, she said, “*I increased my knowledge a lot. Actually, a lot of things [in the past] happened but due to a lack of knowledge I thought it was normal, but now I know that when I see something in a pregnant woman, it could be a complication… I learned a lot and I now have a lot of knowledge.”* She described how POM supported her confidence in being able to advise and inject MgSo4 to women at risk of adverse outcomes associated with pre-eclampsia in her community, “*I didn't ask for help from any nurses since the POM ordered it (MgSo4) and I knew that somehow if the POM orders it, I have to do it…there were no complications*.”

Among male CHWs, simple responses like Alberto's were more frequent. He said, “*The community liked what it (the POM app) did.”* Alberto is a 58-year-old CHW with upper primary school education working in Gaza province for 6 years. As one of the few male CHWs who shared an increase of maternal health knowledge, he reflected, “*Even though I am not assigned as a caregiver, I learned a lot.”* Though Alberto is married and a father of ten children, he did not see himself as a caregiver as men believe that caring is a woman's domain. Alberto reflected that his behavior may have changed as he learned more about pregnancy care and shifted perspectives on males also serving to care for people.

#### Empowerment

Both female and male CHWs reflected that using the mHealth app was empowering and shared that they felt more like a true healthcare professional. While not mentioned by male CHWs, some female CHWs brought up that using POM with local women in their communities during the study helped them realize the importance of their roles and responsibilities (5/31 females vs. 0/12) (see Figure 1).

For example, Luisa, a 59-year-old CHW working in a hard to access area in Maputo province, shared that using POM bolstered her professional status. Even though she reported 17 years of experience working in these communities as a CHW, she said that it was only after working with POM that the communities began seeing her as a healthcare professional. In her words, “*Now the communities see me as a true healthcare professional because I helped many women; I gained new experience I didn't [previously] know… I increased my experience as an advisor and **now I see myself as a true mobilizer** (emphasis added)*. Similarly, Genifa, a married 41-year-old CHW with 21 years of experience working in Gaza province who had completed upper primary school said, “*I felt more important knowing that I'm caring for lives and that I'm saving a lot of people*.”

Male CHWs also shared that they felt like true health professionals but focused on improved interactions with nurses and pregnant women in their communities. An example is Jorge, a 37-year-old, married father of five with 9 years of experience as a CHW in Maputo province. He “*felt like a health professional”* using the POM app. When asked about any changes in behavior, Jorge remarked, “*Every time I checked up on a woman, the nurse would give me the report of what had happened with the sick woman.”* Another example is Feliciano, a 33-year-old, married father of eight who had completed upper primary school and had 4 years of experience as a CHW in Gaza province. When asked to describe any changes to his behavior, Feliciano said, “*I stood firm in doing the work as it should be done, because I knew that it was lives that I was saving.”* He was able to be firm in his counseling because participating in the CLIP trial and using the POM app, “*Gave [him] more knowledge about how to care for pregnant women and how to behave even more in the adverse situations found in the communities with regard to pre-eclampsia and eclampsia.”* Jorge and Felicano's narratives illustrate how male CHWs tended to reflect on improved working relationships with their communities within their conceptualization of feeling like a true health professional, rather than how the communities perceived them, which was more prevalent in narratives from female CHWs.

#### Relationships

Using the mHealth app appears to have influenced relationships in terms of increased value and respect of CHWs by local pregnant women and community members (see Figure 1). The numbers are small and cannot be viewed conclusively, but a gender difference emerged in our study as eighteen of 31 female CHWs said that POM positively influenced their status in the community compared to four of 12 male CHWs.

Narratives from female CHWs had greater focus on how the community gave them more importance, respect and value, that they were seen as more responsible and felt more comfortable in their roles. The following narratives of Joaquina and Paulina both CHWs from Gaza province, highlight how community respect, responsibility and value influenced their interactions with community members and their own sense of worth. Joaquina, a 31-year-old, married with two children who had completed upper primary school and had 3 years as a CHW, reflected on greater care-seeking from women as she became more respected, “*I became more respected. Even on days when there were no visits, the women were looking for information about their condition and with that I gained more strength to work and remain firm.”* This was also seen in Paulina's narrative. Paulina is a married, 29-year-old mother of one with lower secondary school education and three years of experience as a CHW. “*POM gave me more importance, gave me more security, the community gave me more respect and value, came to see [me] as a true health professional.”* She said that, “*[Before the study], I did not know how to measure blood pressure. I did not know the signs of pre-eclampsia/eclampsia but with the use of the POM study, today I am seen as more responsible and it [POM] gave me more value*.”

Isabel's narrative described how the quality of care she provided improved as she felt more comfortable in her role and was more respected as a health care provider in her community. Isabel, a 57-year-old with 11 years of experience and a single mother with five children, said, “*From the moment I had training and began to work, I felt more comfortable. I gained more experience. I became better known as a mother who saves pregnant women,”* when asked about how the use of the POM app affected her role in the community. “*I gained more compassion for caring for more women in my community. I learned a lot about the care required for a pregnancy and what to do when a woman has complications during pregnancy*.”

## Discussion

### Summary of Findings

This secondary qualitative analysis of healthcare worker experiences using the POM app in Mozambique suggested possible gender dimensions around the use of mHealth technologies and provisioning of community healthcare services. Though POM app training requirements and salary support was the same for both male and female CHWs, some potential gender differences were shared in how CHWs experienced the mHealth intervention. Female CHWs often emphasized the positive impacts of POM on their status in the community and elaborate more about community perceptions of their increased value as health care providers than their male counterparts. With increased perceived knowledge and training through the POM app, they gained comfort and confidence with advising women in their communities. As they helped women, they gained status and legitimacy. According to CHWs perceptions, community members began to see the CHWs as true health professionals. In turn, they felt more compassion for pregnant women, which may lead to improved respectful maternity care. While women CHWs were not alone in expressing how their work with POM increased their value and respect among the community, it is of note that female CHWs shared this sentiment more frequently than male CHWs.

### Comparison to Literature

The primary analysis of implementation of POM in rural Mozambique found recognition among nurses of the increased capacity of CHWs in intervention clusters compared to those who did not, as the CHWs who used POM were more confident in their clinical and technological skills to identify women at risk of obstetric emergencies, facilitate early referral of pregnant women at risk of adverse outcomes and support women to attend antenatal care earlier (18). This confirmed CHWs' perceptions that women took their counseling more seriously when guided by POM, which allowed them to provide more clinical maternal health screenings beyond regular health education messages (18). The current secondary analysis builds on the primary findings to highlight potential gender dimensions that may influence and frame interactions between CHWs, community members and mHealth technologies. This is in line with a growing discussion around gender dynamics in digital health particularly in frontline work (2, 21).

Regarding frontline community health work in sub-Saharan Africa in particular, a number of studies noted that gender may not be perceived as an issue for CHW programs at first (22–25). For example, a maternal and child health CHW program from Tanzania found no significant difference between genders in CHW's ability and health promotion activities (22, 23). In Uganda, where there is another mixed-gender CHW program, responsibilities for male and female CHWs were considered to be the same (24). A mixed-gender program in Ethiopia, though the vast majority of CHWs were women, was also described to be guided by national gender mainstreaming policies and purposefully aimed to increase both male and female community members' access to health care (25). In Mozambique, the government encourages recruiting both male and female CHWs, with the aim for gender balance, and perceptions of male and female CHWs were investigated within the CLIP trial before implementing the intervention to understand acceptability. Community members and leaders shared that both male and female CHWs were acceptable, citing that gynecologists at the health facility were often male and women still attended the services as an example. However, this finding failed to take into account the power difference between a gynecologist and CHW and how that may impact the community perception of the role.

A systematic review exploring the impact of gender on CHWs roles and service provisioning in LMICs found that CHW programs working with maternal and child health tend to work within existing gender norms such as the view of women as better caregivers (26), utilizing what some have termed a “women-to-women” approach (27). Studies from Tanzania (22), Uganda (24), Ethiopia (26, 27), Burkino Faso (28) and Kenya, Mozambique, Malawi as well as in Indonesia and Bangladesh (26) found that female CHWs are seen to be more effective at encouraging women to use maternity health services and women may be more comfortable to speak with female CHWs about maternal and child health issues. Male CHWs face barriers to visiting women inside their homes, as found in Mozambique, Malawi (26) and Tanzania (22). Additionally, female CHWs are seen as more available to offer services in the community. A Ugandan study noted that male CHWs may be away from their homes and communities for income generating work while female CHWs were more frequently available in their communities (24). These reflect gender norms and constraints where women are meant to stay closer to home and provide care with more limited ability to generate income, which influence female CHW experiences as illustrated by a narrative of a female CHW from central Mozambique (29). In their study on motivation for becoming a CHW, Maes et al. documented stereotypes that caregiving is women's work as well as limited formal employment opportunities for women, particularly as she had separated from her husband and was the primary source of support for her children (29). Maes et al. revealed that incentives for CHWs in Mozambique were inconsistently provided but she had hoped that her experience would translated into steady, formal employment (29). A study from Zambia also found that women were more likely to join an HIV volunteer program because they wanted “to receive things and allowances”, “get a paying job” or because they “have no job” in comparison with men (30).

These gendered norms and constraints experienced by CHWs speak to a conceptual framework of factors affected by gender that includes individual (family support and safety/security), community (acceptability and mobility) and health systems (career advancement and remuneration) levels (26). While the influence of gender on workplace violence for female health workers has been found elsewhere within the sub-Saharan African context (26, 27, 31, 32) as are issues of remuneration and career advancement (27, 33, 34), these did not emerge in our study that focused on experiences of implementing an mHealth intervention to improve maternal health at the community level. Rather, what emerged was a more subtle discussion about community perceptions of female CHWs in terms of increased value and respect as professional health providers within their communities.

While working within existing gender norms around caregiving may support female CHWs' health promotion work in maternal and child health issues and support the comfort of women to speak to another woman, gender norms may also lend to questioning the professionalism of female CHWs more than for their male counterparts. There is some evidence from Rwanda where a health worker survey with physicians, nurses, midwives, and social workers found negative stereotypes of female health workers as unwilling to speak up, indecisive, and less competent with clinical activities (31). These issues have been widely explored in South Asia, where Mumtaz et al. highlight that the social construction of women as primarily housewives meant that they had to work harder than men to be accepted as health workers (35). Though it is important to note the different context between Pakistan where there is more explicit gender segregation and our study in Mozambique, the idea that gender norms initially support the recruitment of women as CHWs but may hinder community perceptions of them as health workers may be relevant. Empowerment themes that the community gave female CHWs more importance, respect and value and that they were seen as more responsible health professionals with the use of POM may suggest some of the ways that CHWs professional roles were undervalued previously. That these sentiments were expressed more frequently among female CHWs may suggest that gender shaped the negotiation of professional status with the communities and may contribute to why female CHWs in this study more frequently mentioned their increased status as one of the key impacts of participating in the study. Though the process to become a CHW in Mozambique includes 4 months of training and marked by a graduation ceremony, narratives especially from female CHWs in our study suggested that it took further training and performance of mHealth procedures with clinical activities for many to feel recognized as a healthcare worker in their communities. More research is needed to further explore these topics, particularly within contexts where gender is not seen to be a concern at first such as mentioned in some CHW programs in sub-Saharan Africa (22–25).

### Strengths and Limitations

The exploratory approach of this secondary analysis contains a number of limitations. First, gender was not an explicit focus of the health care worker evaluation design and emerged as a topic of further investigation following the primary analysis. Consequently, issues were spontaneously brought up by respondents rather than systematically queried. Secondly, though data saturation in qualitative research has been indicated to be reached after the 12th interview (17), our gender analysis is limited by the small sample size, particularly of male CHWs who used POM. For example, our research potentially indicated that some male CHWs may have experienced attitude changes around caregiving, though the small sample of male CHWs in the gender analysis limits our ability to explore in depth. Additionally, there were unbalanced samples of male and female CHWs. However, given that frontline health worker at the community and primary health level is often over-represented by women (4), the higher ratio of female CHWs in our study speak to realistic representation within local health systems. Furthermore, while narrative excerpts did not appear to be differentiated by educational level, our gender analysis is limited by missing baseline educational level among a substantial portion of our sample (28%). Lastly, the study was limited by its cross-sectional design that only evaluated post-intervention and a reliance on self-report by CHWs, though the meanings that participants construct of an experience is emphasized within a constructivist approach (15). While the current exploratory study highlights possible gender dimensions around the use of mHealth technologies in the provisioning of community healthcare services from the perspective of CHWs, more research is needed from community members to understand their perspectives.

A strength of the exploratory approach of the current study is to highlight potential areas of future exploration where there are current gaps in the literature, such as the influence of gender on community perceptions of CHW professionalism. Future research is recommended to explore male CHW empowerment, highlighting ways their involvement may be gender-transformative rather than fitting into existing gender norms. Additionally, future research can further investigate CHWs' education as a potential influencing factor and if baseline educational level was associated with gender. Another area of potential research around gendered community health work is further investigation into the impact of technology use and associated increased perception of skill on future plans for employment. In future, we recommend expanding on lessons learned in this study to understand the role technology plays in shaping how CHWs feel their current work supports their career advancement.

## Conclusion

The emphasis female CHWs in our study placed on certain topics, such as increased respect and status as a true health professional, suggest the influence of gender in the health systems and communities they live and work in. The mHealth intervention was valued beyond the technology itself because health workers perceived that it strengthened clinical capacities and community perceptions of their value, which may have been especially appreciated by the female CHWs. Their stories share tangible ways that investing in community health workers and building their capacity for clinical care can have far ranging gender transformative results. Understanding the gendered dimensions of community health work and investing in frontline workers to develop their clinical capacities can play a valuable part in reducing gender biases in health systems and more research is needed to understand the perspectives of CHWs and the communities they work in to substantiate our exploratory findings.

## Data Availability Statement

De-identified raw data supporting the conclusions of this article will be made available by the authors, without undue reservation.

## Ethics Statement

The studies involving human participants were reviewed and approved by the CISM Institutional Review Board in Mozambique (CIBS- CISM/08/2013) and the UBC C&W Research Ethics Board in Canada (H12-00132). The patients/participants provided their written informed consent to participate in this study.

## Author Contributions

PD, ES, KM, and LM: conceptualized the trial and components of the intervention. BP, ES, and KM: conceptualized the health worker evaluation. HB, AV, SS, BP, and MV: supported the implementation of the trial and health worker evaluation. MWK: conceptualized and performed this secondary analysis. MWK and BP: wrote the first draft of the manuscript. All authors provided feedback and review of the manuscript.

## Funding

This trial received no specific research funding but salary support for BP to facilitate study design and implementation was awarded through a CIHR Fellowship (CIHR-IRSC:02070000821). The CLIP trial was funded by the University of British Columbia, a grantee of the Bill & Melinda Gates Foundation (PRE-EMPT initiative, Grant Number OPP1017337). Following input into trial design, the Gates Foundation had no role in data collection, analysis, or interpretation, or writing of the report.

## Conflict of Interest

The authors declare that the research was conducted in the absence of any commercial or financial relationships that could be construed as a potential conflict of interest.

## Publisher's Note

All claims expressed in this article are solely those of the authors and do not necessarily represent those of their affiliated organizations, or those of the publisher, the editors and the reviewers. Any product that may be evaluated in this article, or claim that may be made by its manufacturer, is not guaranteed or endorsed by the publisher.
