# Supplementary material for: How Gender Influenced the Experience of Using a mHealth Intervention in Rural Mozambique: Secondary Qualitative Analysis of Community Health Worker Survey Data
Source: Front Glob Womens Health. 2022 Feb 24;3:661000. doi: 10.3389/fgwh.2022.661000 (PMC8907823; doi:10.3389/fgwh.2022.661000)
Supplement: Supplementary file 1 [file Data_Sheet_1.docx]

**Supplementary tables**

**Table S1 CLIP Mozambique Working Group**

| **CLIP Mozambique Working Group** | |
| --- | --- |
| **First and middle names** | **Last names** |
| Felizarda | Amose |
| Ana Ilda | Biz |
| Rogério | Chiaú |
| Silvestre | Cutana |
| Paulo | Filimone |
| Marta | Macamo |
| Sónia | Maculuve |
| Ernesto | Mandlate |
| Analisa | Matavele |
| Sibone | Mocumbi |
| Dulce | Mulungo |
| Zefanias | Nhamirre |
| Ariel | Nhancolo |
| Cláudio | Nkumbula |
| Vivalde | Nobela |
| Rosa | Pires |
| Faustino | Vilanculo |
| Rahat N | Qureshi |
| Sana | Sheikh |
| Zahra | Hoodbhoy |
| Imran | Ahmed |
| Amjad | Hussain |
| Javed | Memon |
| Farrukh | Raza |
| Mrutunjaya B | Bellad |
| Shivaprasad S | Goudar |
| Ashalata A | Mallapur |
| Shashidhar G | Bannale |
| Umesh S | Charantimath |
| Keval S | Chougala |
| Richard J | Derman |
| Vaibhav B | Dhamanekar |
| Narayan V | Hoonungar |
| Anjali M | Joshi |
| Namdev A | Kamble |
| Chandrasekhar | Karadiguddi |
| Geetanjali M | Katageri |
| Avinash J | Kavi |
| Gudadayya S | Kengapur |
| Bhalachandra S | Kodkany |
| Uday S | Kudachi |
| Sphoorthi S | Mastiholi |
| Geetanjali I | Mungarwadi |
| Umesh Y | Ramadurg |
| Amit P | Revankar |
| Olalekan O | Adetoro |
| John O | Sotunsa |
| Sharla K | Drebit |
| Chirag | Kariya |
| Mansun | Lui |
| Diane | Sawchuck |
| Ugochi V | Ukah |
| Mai-Lei Woo | Kinshella |
| Shafik | Dharamsi |
| Guy A | Dumont |
| Tabassum | Firoz |
| Ana Pilar | Betrán |
| Susheela M | Engelbrecht |
| Veronique | Filippi |
| William A | Grobman |
| Marian | Knight |
| Ana | Langer |
| Simon A | Lewin |
| Gwyneth | Lewis |
| Craig | Mitton |
| Nadine | Schuurman |
| James G | Thornton |
| France | Donnay |
| Kelly | Pickerill |
| **CLIP Trial Working Group** | |
| Esperança Sevene, Eusébio Macete, Khátia Munguambe, Charfudin Sacoor, Anifa Vala, Helena Boene, Felizarda Amose, Rosa Pires, Zefanias Nhamirre, Marta Macamo, Rogério Chiaú, Analisa Matavele, Faustino Vilanculo, Ariel Nhancolo, Silvestre Cutana, Ernesto Mandlate, Salésio Macuacua, Quinhas Fernandes, Rosa Marlene Cuco, Cassimo Bique, Sibone Mocumbi, Emília Gonçálves, Sónia Maculuve, Ana Ilda Biz, Dulce Mulungo, Orvalho Augusto, Paulo Filimone, Vivalde Nobela, Corsino Tchavana, Cláudio Nkumbula  Jeffrey Bone, Dustin T Dunsmuir, Sharla K Drebit, Chirag Kariya, Mai-Lei Woo Kinshella, Tang Lee, Jing Li, Mansun Lui, Beth A Payne, Kelly Pickerill, Diane Sawchuck, Sumedha Sharma, Domena K. Tu, Marianne Vidler, Ugochi V Ukah, Laura A Magee, Peter von Dadelszen | |
| **CLIP Trial Adjudication Committee** | |
| Nafissa Osman, Cassimo Bique, Natercia Fernandes, Betuel Sigauque Raquel Gonzalez | |
| **CLIP Trial Steering Committee** | |
| J Mark Ansermino, Ana Pilar Betrán, Richard Derman, Shafik Dharamsi, France Donnay, Sharla Drebit, Guy Dumont, Susheela M. Engelbrecht, Veronique Fillipi, Tabassum Firoz, William Grobman, Marian Knight, Ana Langer, Simon Lewin, Gwyneth Lewis, Craig Mitton, Nadine Schuurman, Andrew H Shennan, Joel Singer, Jim Thornton, Hubert Wong | |
| **CLIP Trial Executive Committee** | |
| Olalekan Adetoro, Mrutunjaya M Bellad, Zulfiqar Bhutta, Peter von Dadelszen, Shivaprasad S Goudar, Jerker Liljestrand, Laura A Magee, Ashalata Mallapur, Khátia Munguambe, Beth Payne, Rahat Qureshi, Charfudin Sacoor, Esperança Sevene, Sumedha Sharma, John Obafemi Sotunsa, Marianne Vidler | |
| **CLIP Data Safety and Monitoring Board (DSMB**) | |
| Romano Nkumbwa Byaruhanga, Brian Darlow, Eileen Hutton, Mario Merialdi, Lehana Thabane | |

| **Table S2 Standards for Reporting Qualitative Research (SRQR)** |
| --- |
| <http://www.equator-network.org/reporting-guidelines/srqr/> |
| \|  \|  \|  \| \| --- \| --- \| --- \| \| **Title and abstract** \| \| **Page/line no(s).** \| \|  \| **Title** - Concise description of the nature and topic of the study Identifying the study as qualitative or indicating the approach (e.g., ethnography, grounded theory) or data collection methods (e.g., interview, focus group) is recommended \| Pg 1 \| \|  \| **Abstract** - Summary of key elements of the study using the abstract format of the intended publication; typically includes background, purpose, methods, results, and conclusions \| Pg 1-2 \| \|  \|  \|  \| \| **Introduction** \| \|  \| \|  \| **Problem formulation** - Description and significance of the problem/phenomenon studied; review of relevant theory and empirical work; problem statement \| Pg 2-3 \| \|  \| **Purpose or research questio**n - Purpose of the study and specific objectives or questions \| Pg 3 \| \|  \|  \|  \| \| **Methods** \| \|  \| \|  \| **Qualitative approach and research paradigm** - Qualitative approach (e.g., ethnography, grounded theory, case study, phenomenology, narrative research) and guiding theory if appropriate; identifying the research paradigm (e.g., postpositivist, constructivist/ interpretivist) is also recommended; rationale** \| Pg 3 \| \|  \| **Researcher characteristics and reflexivity** - Researchers’ characteristics that may influence the research, including personal attributes, qualifications/experience, relationship with participants, assumptions, and/or presuppositions; potential or actual interaction between researchers’ characteristics and the research questions, approach, methods, results, and/or transferability \| Pg 3 \| \|  \| **Context** - Setting/site and salient contextual factors; rationale** \| Pg 3 \| \|  \| **Sampling strategy** - How and why research participants, documents, or events were selected; criteria for deciding when no further sampling was necessary (e.g., sampling saturation); rationale** \| Pg 3 \| \|  \| **Ethical issues pertaining to human subjects** - Documentation of approval by an appropriate ethics review board and participant consent, or explanation for lack thereof; other confidentiality and data security issues \| Pg 4-5 \| \|  \| **Data collection methods** - Types of data collected; details of data collection procedures including (as appropriate) start and stop dates of data collection and analysis, iterative process, triangulation of sources/methods, and modification of procedures in response to evolving study findings; rationale** \| Pg 3 \| \|  \| **Data collection instruments and technologies** - Description of instruments (e.g., interview guides, questionnaires) and devices (e.g., audio recorders) used for data collection; if/how the instrument(s) changed over the course of the study \| Pg 3 \| \|  \| **Units of study** - Number and relevant characteristics of participants, documents, or events included in the study; level of participation (could be reported in results) \| Pg 3 \| \|  \| **Data processing** - Methods for processing data prior to and during analysis, including transcription, data entry, data management and security, verification of data integrity, data coding, and anonymization/de-identification of excerpts \| Pg 4 \| \|  \| **Data analysis** - Process by which inferences, themes, etc., were identified and developed, including the researchers involved in data analysis; usually references a specific paradigm or approach; rationale** \| Pg 4 \| \|  \| **Techniques to enhance trustworthiness** - Techniques to enhance trustworthiness and credibility of data analysis (e.g., member checking, audit trail, triangulation); rationale** \| Pg 4 \| \|  \|  \|  \| \| **Results/findings** \| \|  \| \|  \| **Synthesis and interpretation** - Main findings (e.g., interpretations, inferences, and themes); might include development of a theory or model, or integration with prior research or theory \| Pg 5-7 \| \|  \| **Links to empirical data** - Evidence (e.g., quotes, field notes, text excerpts, photographs) to substantiate analytic findings \| Pg 5-7 \| \|  \|  \|  \| \| **Discussion** \| \|  \| \|  \| **Integration with prior work, implications, transferability, and contribution(s) to the field -** Short summary of main findings; explanation of how findings and conclusions connect to, support, elaborate on, or challenge conclusions of earlier scholarship; discussion of scope of application/generalizability; identification of unique contribution(s) to scholarship in a discipline or field \| Pg 8-9 \| \|  \| **Limitations** - Trustworthiness and limitations of findings \| Pg 9-10 \| \|  \|  \|  \| \| **Other** \| \|  \| \|  \| **Conflicts of interest** - Potential sources of influence or perceived influence on study conduct and conclusions; how these were managed \| Pg 10 \| \|  \| **Funding** - Sources of funding and other support; role of funders in data collection, interpretation, and reporting \|  \| |
|  |

**Table S3. Impact of using the mHealth app by gender**

*Total respondents reflects the number of CHWs who brought up the given theme within their response.*

|  | | **Overall CHWs (n=43)** | **Female CHWs (n=31)** | **Male CHWs (n=12)** |
| --- | --- | --- | --- | --- |
| Perceived knowledge | Total respondents | 23 (54%) | 19 (61%) | 4 (33%) |
|  | Gained knowledge in general | 10 (23%) | 9 (29%) | 1 (8%) |
|  | Learned about pre-eclampsia, urine testing and how to measure blood pressure | 9 (21%) | 7 (22%) | 2 (17%) |
|  | Learned more about pregnancy care and complications | 7 (16%) | 6 (19%) | 1(8%) |
|  | Learned to work with community | 3 (7%) | 1 (3%) | 2 (17%) |
| Self-efficacy | Total respondents | 21 (49%) | 16 (52%) | 5 (42%) |
|  | Ability to inject MgSo4 | 11 (26%) | 7 (23%) | 4 (33%) |
|  | Increased confidence and skill in delivering services | 11 (26%) | 10 (32%) | 1(8%) |
|  | Using clinical assessment and treatment skills learned | 4 (9%) | 3 (10%) | 1 (8%) |
| Empowerment | Total respondents | 15 (35%) | 11 (36%) | 4 (33%) |
|  | Realization of ability to help women and to save lives | 8 (19%) | 6 (19%) | 2 (17%) |
|  | Changes in self-identity | 6 (14%) | 2 (7%) | 4 (33%) |
|  | Realization of importance of roles and responsibilities | 5 (12%) | 5 (16%) | 0 (0%) |
| Relationships | Total respondents | 29 (67%) | 22 (71%) | 8 (67%) |
|  | Increased value and respect in CHWs by pregnant women and community members | 22 (51%) | 18 (58%) | 4 (33%) |
|  | Improved quality of care with women | 8 (19%) | 5 (16%) | 3 (25%) |
|  | Improved relationships with nurses | 4 (9%) | 2 (7%) | 2 (17%) |
